# The mechanistic role of neutrophil lymphocyte ratio perturbations in the leading non communicable lifestyle diseases

**DOI:** 10.12688/f1000research.123245.1

**Published:** 2022-08-19

**Authors:** Monalisa Biswas, Renuka Suvarna, Vimal Krishnan S, Tom Devasia, Vijetha Shenoy Belle, Krishnananda Prabhu

**Affiliations:** 1 Department of Biochemistry, Kasturba Medical College, Manipal, Manipal Academy of Higher Education, Manipal, Karnataka, 576104, India; 2 Division of Ayurveda, Center for Integrative Medicine and Research, Manipal, Manipal Academy of Higher Education, Manipal, Karnataka, 576104, India; 3 Department of Emergency Medicine, Kasturba Medical College, Manipal, Manipal Academy of Higher Education, Manipal, Karnataka, 576104, India; 4 Department of Cardiology, Kasturba Medical College, Manipal, Manipal Academy of Higher Education, Manipal, Karnataka, 576104, India

**Keywords:** Neutrophil-lymphocyte ratio, non communicable diseases, inflammation, type 2 diabetes mellitus, coronary artery disease, chronic obstructive pulmonary disease

## Abstract

Inflammation plays a critical role in the development and progression of chronic diseases like type 2 diabetes mellitus, coronary artery disease, and chronic obstructive pulmonary disease. Inflammatory responses are indispensable for pathogen control and tissue repair, but they also cause collateral damage. A chronically activated immune system and the resultant immune dysregulation mediated inflammatory surge may cause multiple negative effects, requiring tight regulation and dampening of the immune response to minimize host injury.  While chronic diseases are characterized by systemic inflammation, the mechanistic relationship of neutrophils and lymphocytes to inflammation and its correlation with the clinical outcomes is yet to be elucidated. The neutrophil to lymphocyte ratio (NLR) is an easy-to-measure laboratory marker used to assess systemic inflammation. Understanding the mechanisms of NLR perturbations in chronic diseases is crucial for risk stratification, early intervention, and finding novel therapeutic targets. We investigated the correlation between NLR and prevalent chronic conditions as a measure of systemic inflammation. In addition to predicting the risk of impending chronic conditions, NLR may also provide insight into their progression. This review summarizes the mechanisms of NLR perturbations at cellular and molecular levels, and the key inflammatory signaling pathways involved in the progression of chronic diseases. We have also explored preclinical studies investigating these pathways and the effect of quelling inflammation in chronic disease as reported by a few *in vitro*, *in vivo* studies, and clinical trials.

## 1. Introduction

Inflammation is an evolutionary physiological defense reaction in response to injurious or noxious external stimuli. ^1^ Inflammation aims to maintain a stable and constant internal environment and protect against detrimental tissue injury stimulus by countering the infectious agent/threatening source and stimulating healing. ^2^ Inflammatory responses are triggered by innate activations, and the immune system responds to the trigger via activation of inflammatory cascades supposedly orchestrated to eliminate the root cause of the inflammation. ^1^^,^^2^ Acute inflammation is an essential and indispensable rapid response to pathogenic attacks or environmental injuries and aids the body to eliminate the threatening stimuli. ^1^ However, inflammation is a double-edged sword and in situations where acute inflammation fails to resolve, it evolves into a sustained underlying chronic inflammation and a resultant chronically activated immune system that manifests its catastrophic effects on healthy tissues. ^2^^,^^3^

The process of achieving chronicity and its progression is believed to be influenced by multiple environmental, genetic, and behavioural variables resulting in numerous routes of inception and progression of inflammation-associated chronic non-communicable lifestyle diseases such as type 2 diabetes, atherosclerosis, ischemic and renal disorders, cancer metastasis, etc. Lifestyle variables such as unhealthy diet, smoking, alcoholism, obesity, sedentary mode, and predisposing genetic variables (familial hypercholesterolemia/hypertriglyceridemia, hypersensitivity, autoimmunity) exert exacerbating effect on chronic low-grade inflammation and trigger the initiation and progression of vascular lesions. ^3^

According to the World Health Organisation, diabetes, cardiovascular diseases (such as heart attacks and stroke), cancers, and chronic respiratory diseases (such as chronic obstructive pulmonary disease and asthma) are the four major threatening noncommunicable diseases (NCDs) that account for approximately 41 million deaths annually (71% of all deaths globally) with 85% being “premature” deaths. Cardiovascular diseases account for most NCD deaths, or 17.9 million people annually, followed by cancers (9.3 million), respiratory diseases (4.1 million), and diabetes (1.5 million). The role of inflammation in the inception and progression of the NCD cluster diseases is undebatable and the disease course is further impacted by several inflammation-associated modifiable (diet, obesity, substance abuse, physical inactivity) and metabolic (hypertension, hyperglycemia, hyperlipidaemia, obesity) risk factors which makes it imperative to trace the mechanistic paths of inception and progression of inflammation and explore inflammation check as a robust preventive and therapeutic strategy in management and alleviation of these NCDs. ^4^

Chronic inflammation triggers cytokine-mediated activation of leukocytes (neutrophils and lymphocytes) and endothelial cells, through a complex network of immunological cascades. ^1^ The neutrophil to lymphocyte ratio (NLR) has emerged as a novel haematological indicator of systemic inflammation and is being widely explored across medical disciplines due to its advantage of being an easily accessible, cheap, and apparently reliable biomarker of inflammatory and immune cell recruitment status in infectious and non-infectious disease scenarios. NLR represents a dynamic index of cross interactions and perturbations between innate (neutrophils) and adaptive cellular immunity (lymphocytes) and is influenced by various pathological and lifestyle factors. NLR has been claimed to be a sensitive indicator of inflammation (infective as well as noninfective), diagnosis/stratification, prediction of the clinical course, and prognosis. The NLR changes precede the acute clinical presentations by several hours and may be perceived as early warning signals of the underlying ongoing pathological progression which makes NLR a novel prospective marker of immune activation, and a reliable index of systemic inflammation. NLR aids an in-depth understanding of inflammation biology, coupling, and antagonism between innate and adaptive immunity, and its implications in diseases. ^5^

The present review aims to explore and gain an understanding of the pathological mechanism which triggers NLR perturbations in the three leading NCDs (type 2 DM, coronary artery disease, and chronic obstructive pulmonary disease), the reliability and validity of this marker in assessing disease severity, and progression and thus paves gateway for exploration of novel and adjuvant inflammation associated screening, risk stratification as well as therapeutic targets of the candidate NCDs.

## 2. Type 2 Diabetes Mellitus (T2DM)

T2DM is classically characterized by a chronic low-grade systemic inflammation, with disrupted circulating levels of inflammatory cytokines which not only contribute to the vicious progression of hyperglycemia and insulin resistance but also pave the silent yet catastrophic gateway to the long-term end-organ vascular complications of diabetes mellitus. ^6^

### 2. A) Neutrophils in T2DM

Neutrophil perturbations and dysfunctions play central and regulatory roles in the inception, and progression of type 2 diabetes mellitus and its secondary complications including increased susceptibility to opportunistic infections. ^7^^,^^8^

**Figure 1.**
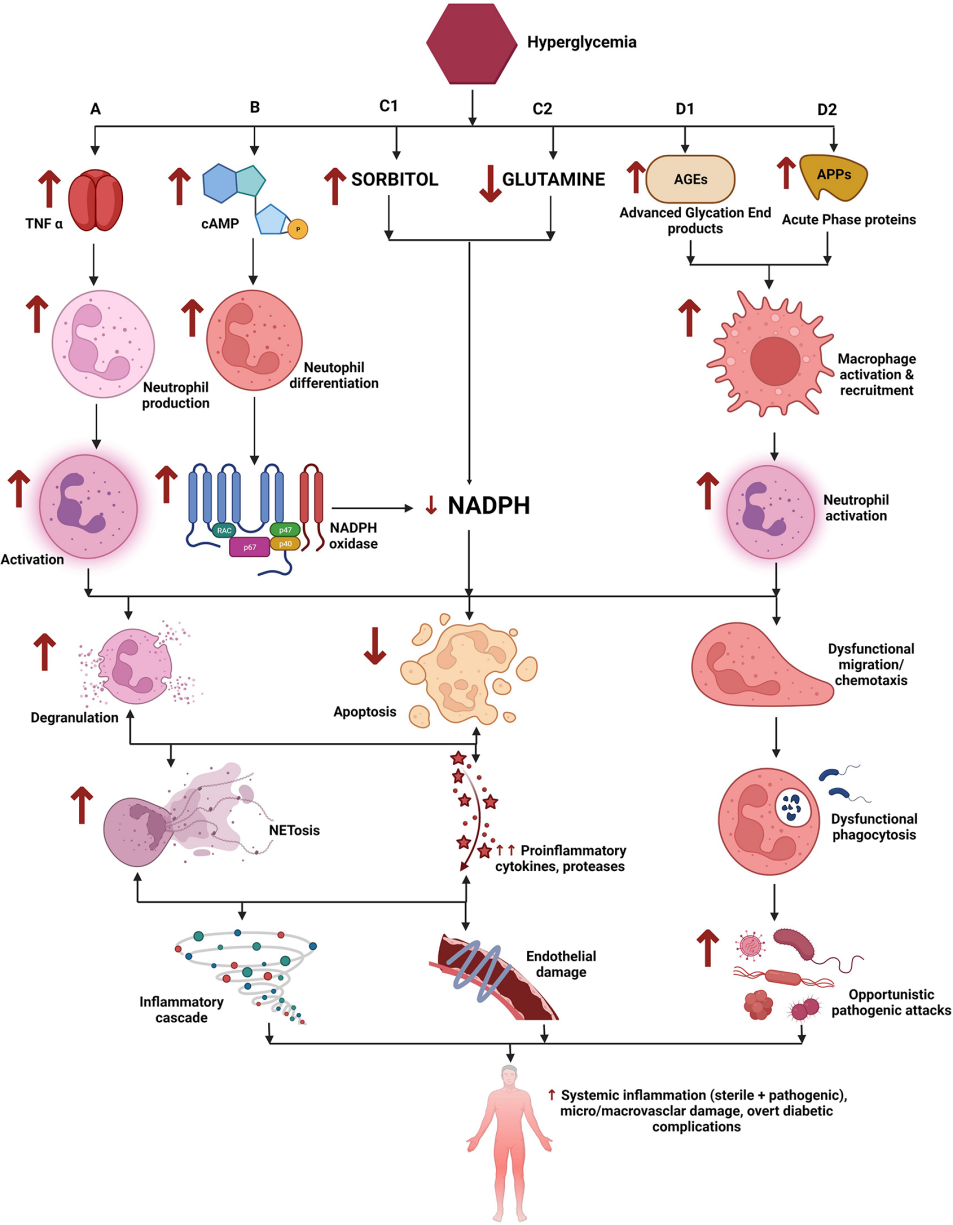
Role of neutrophils in Type 2 Diabetes Mellitus (T2DM). A) Hyperglycemia leads to enhanced stimulation of TNF alpha secretion which promotes neutrophil generation and subsequent neutrophil activation. B) Hyperglycemia increases intracellular cAMP which leads to increased generation of inflammatory mediators and reactive oxygen species (ROS). C1) Hyperglycemia activates the polyol pathway and leads to increased sorbitol generation. C2) Decreased glutamine has been implicated in hyperglycemia. B + C1 + C2 leads to exaggerated utilization and thus depletion of cellular NADPH. D1, D2) Hyperglycemia triggers the formation of advanced glycation end products (AGEs) and the release of acute-phase proteins (APPs) which in turn causes increased neutrophil activation. Exaggerated neutrophil activation and decreased cellular NADPH concentrations in the background of an inflammatory microenvironment, leads to dysfunctional/hyperactivated neutrophil which manifest increased degranulation, resistance to apoptosis, and dysfunctional chemotactic abilities. This cascade results in widespread systemic inflammation leading to micro and macrovascular complications of diabetes mellitus. Note: ↑ indicates increased levels while ↓ refers to decreased levels/concentrations. This figure is an original figure produced by the authors for this review article and has been filed for copyright with the Copyright Office, Government of India.

The mechanistic pathology and the role of the major neutrophilic alterations observed in T2DM include:

**2.A.i. Neutrophilia (** **Figure 1** **):** Numerous scientific studies have shown increased circulating levels of neutrophils in T2DM patients when compared to healthy individuals. A study has reported that peripheral neutrophil counts are shown to increase progressively from T1DM (type 1 diabetes mellitus), LADA (latent autoimmune diabetes in adults) to T2DM and correlate well with residual β-cell function. ^9^ Experimental evidence has shown that insulin resistance and hyperglycemia itself contributes to increased neutrophil count. ^10^ However, neutrophil count may be affected by multiple factors including systemic inflammation, hypercholesterolemia, ageing, and sleep disorders. ^11^

**2.A.ii. NETosis (** **Figure 1** **):** Neutrophil extracellular traps (NETs) have been implicated in the pathogenesis of T2DM. Literature evidence are replete with claims of exaggerated NET formation in T2DM and its micro and macrovascular complications. A study showed significantly elevated levels of NETs in patients with diabetes and diabetic retinopathy. ^12^ However, NET production is believed to be triggered by NADPH (nicotinamide adenosine dinucleotide phosphate) oxidase activation secondary to high circulating glucose concentrations. NET formation is also thought to be triggered by cytokine stimulation and the grade/extent of hyperglycemia. ^13^

Studies have also shown evidence that NETs correlate with HbA1c and proinflammatory cytokines like IL-6 and TNFα (tumour necrosis factor alpha). Further, evidence shows that restoration of elevated circulating NETs could need a longer duration of treatment than controlling blood sugar emphasizing that NETs and NETosis may be attributed more to the chronicity of inflammation rather than hyperglycemia. ^14^ Multiple studies proved that NETs play a key role in the progression of various micro and macrovascular diabetic complications. ^15^ A study reported that neutrophils isolated from T2DM patients as well as mice were primed to produce NETs, diabetic mice manifested increased levels of citrullinated H3 and showed delayed wound healing, while wound healing was accelerated by the addition of DNase 1, an inhibitor of NETosis. ^16^

**2.A.iii. Abnormal neutrophil activation (** **Figure 1** **):** A study on comprehensive transcriptome analysis of neutrophils reported that the differentially expressed genes are involved in the activation of leukocytes and T lymphocytes, cytokine production, and adaptive immune system function which could explain the observed neutrophil dysfunction and the resultant immune perturbations seen in chronic and uncontrolled T2DM. ^17^ A study investigated the altered release of inflammatory cytokines (by neutrophils and monocytes) of diabetics and found that neutrophils of diabetics released higher folds of these cytokines than healthy subjects in basal as well as in LPS (lipopolysaccharide) stimulated cultures. The authors concluded that this alteration in cytokine mechanism could explain the observed neutrophil dysfunction in diabetics. It is hypothesized that the chronic stimulation of cytokine production by diabetic neutrophils may be triggered by AGE (advanced glycation end products)-associated protein modifications, increase in intracellular ionic calcium, activation of phagocytes, and the downstream stimulation of pro-inflammatory cytokine transcription and secretion. ^18^

**2.A.iv. Dysfunctional neutrophils/Neutrophil apoptosis (** **Figure 1** **):** Studies have reported defective chemotactic, phagocytic, anti-microbial, and secretory functions (lysosomal enzymes, hydrogen peroxide, etc) in neutrophils of patients/animals with T2DM while lowering blood glucose levels have been reported to bring about improvement in neutrophil activity. ^7^^,^^8^ A study reported significantly lower neutrophil chemotactic activity in diabetics when compared to healthy individuals. ^19^

An inflammatory response induces leukocytes/neutrophil extravasation which involves firm adherence to the vasculature prior to their migration to the injured tissues. This involves the interaction of cell adhesion glycoproteins which mediate leukocyte rolling and adherence and one plausible explanation of neutrophil/leukocyte dysfunction is the downregulation of these cell adhesion glycoproteins (related to structural modifications due to AGEs). Also, alpha-1-acid glycoprotein (AGP), an acute-phase protein, usually elevated in T2DM has been implicated in the impairment of neutrophil migration and chemotaxis in animal models. ^7^ Further, resistin, a cysteine-rich protein hormone secreted by macrophages and elevated in T2DM patients, has shown to decrease fMLP (proinflammatory peptide formyl-methionyl-leucyl-phenylalanine)-induced neutrophil chemotaxis in vitro (inhibition of PI3K [phosphatidylinositol-3 kinase] pathway activation) and PMA (phorbol 12-myristate 13-acetate)/Ecoli induced oxidative burst.

Neutrophils utilize high amounts of glutamine for their metabolic functioning. Studies have shown a significant decrease in glutamine oxidation and glutaminase activity in neutrophils of diabetic rats. ^20^ Glutamine has been implicated to regulate NADPH production, NADPH oxidase activity, and pathogen-stimulated ROS (reactive oxygen species) generation in neutrophils. ^21^ Decreased glutamine oxidation can therefore lead to altered neutrophil function as well as premature neutrophil apoptosis. ^21^^,^^22^

**2.A.v. Advanced Glycation End products (AGEs) (** **Figure 1** **):** Hyperglycemia is known to trigger the formation of AGEs through a spontaneous nonenzymatic equilibration process, which accumulates in the vascular smooth muscle, and phagocytic cells. These alterations are implicated in the micro and macrovascular complications of T2DM and may also interfere with leukocyte and neutrophil function. A study reported altered leukocyte membrane fluidity, due to AGEs, in diabetic rats and attributed this change to the altered leukocyte migration. ^23^ Sustained stimulation by AGEs is shown to cause altered neutrophil extravasation through endothelial layers, induced by impaired chemotactic peptide formyl-Met-Leu-Phe. ^24^

**2.A.vii. Polyol pathway (** **Figure 1** **):** The polyol pathway has been implicated in neutrophil dysfunction. ^25^ It is hypothesized that increased production of sorbitol by the polyol pathway increases intracellular osmolarity and depletes NADPH leading to perturbed endothelial cell function and dysfunction in leukocyte-endothelial cell interactions. ^20^^,^^26^

**2.A.vii. Oxidative stress:** Multiple human and animal studies have shown increased expression as well as circulating concentrations of reactive oxygen species, while diminished pathogen stimulated production of ROS in T2DM. Further, studies have also shown restoration of inducible NO (nitric oxide) to normal concentration and expression after three-day treatment with NPH (Neutral Protamine Hagedorn) insulin, in alloxan-induced diabetic rats. cGMP (cyclic guanosine monophosphate) has been implicated in these findings since neutrophil cGMP concentrations were found to be elevated in rats after ten-day of exposure to alloxan. ^20^^,^^27^

### 2. B) Lymphocytes in T2DM

Recent advances in diabetes research suggest a major contribution of the adaptive immune system in the pathogenesis and progression of T2DM, with T cell perturbations playing a pivotal role in the attenuated immune activation in T2DM. ^28^

Major lymphocyte perturbations observed include:

**2.B.i. Lymphocytopenia:** A study on 37 healthy subjects explored the effects of standardized hyperglycemia on immune cells and reported a significant decrease in total white blood cells in response to short-term hyperglycemia when compared to placebo. ^29^ Studies have reported that T2DM patients demonstrate insufficient lymphocyte proliferation owing to the decreased expression of IL2 receptors. Another study also reported decreased lymphocyte and increased neutrophil counts in patients with uncontrolled diabetes. ^30^ A study shows that patients with T2DM exhibit elevated intracellular ionic calcium in B cells when compared to normoglycemic healthy controls, the concentration of intracellular ionic calcium significantly correlates with the extent of hyperglycemia, further the B cells of the T2DM participants showed impaired proliferation in response to *Staphylococcus aureus* Cowan I. ^31^

**2.B.ii. Lymphocyte dysfunction:**

**a) Metabolic effects of hyperglycemia:** A study explored the activity of key enzymes of glucose metabolism in rat lymphocytes and reported that hexokinase, G6PD (glucose 6 phosphate dehydrogenase), and citrate synthase showed decreased activity while PFK (phosphofructokinase) showed increased activity in diabetes. These metabolic changes may be responsible for the observed impairment of lymphocyte function in T2DM. ^32^ A study reported that patients with T2DM showed increased circulating levels of insulin (insulin resistance) and decreased percentages of total lymphocytes, CD8 ^+^ T, effector CD4 ^+^ T, and B lymphocytes, while Treg cells were reported to be increased in T2DM. ^33^

**b) Effects due to other metabolic perturbations:** Studies have shown decreased glycolysis and mitochondrial respiration in circulating CD8 ^+^ T cells, impaired cytokine production in CD8 ^+^ PD-1 ^+^ T cells as well as impaired CD8 ^+^ PD-1 ^+^ T cells leading to reduced antigen specific response in T2DM. Based on previous evidences pertaining to tumour microenvironment induced changes in T cells and other studies on T2DM which point towards an exhaustive phenotype, these changes in T cell metabolism and function can most probably be attributed to chronic activation induced T cell exhaustion. ^34^ A study reported that CD26 expression was significantly decreased while ADA activity was significantly increased in T2DM patients which could also explain altered lymphocyte function in T2DM. ^35^

**2.B.iii. Ketoacidosis induced abnormal lymphocyte activation:** Hyperglycaemia induced stress, however, has shown to induce oxidative damage in T lymphocytes. A study reported the in vivo activation of CD4 ^+^ and CD8 ^+^ lymphocytes and de novo expression of IGF 1 (insulin like growth factor), and IL 2, most probably associated with increased TBARs (thiobarbituric acid reactive substances), and dichlorofluorescien. Thus, DKA (diabetic ketoacidosis) activates T lymphocytes. ^36^ Another study reported that T lymphocytes isolated from diabetic ketoacidosis patients show activated CD4 ^+^ and CD8 ^+^ T cells in addition to increased circulating concentrations of pro-inflammatory cytokines. ^37^

### 2. C) NLR in T2DM

Studies have shown that NLR is not only an inflammatory marker but also shows predictive, diagnostic as well as prognostic potential for secondary complications of T2DM including atherosclerosis, early diabetic nephropathy, retinopathy, and foot ulcers. A study reported that elevated NLR was strongly associated with glucose intolerance and insulin resistance. ^38^

A study on 330 T2DM patients reported that NLR was significantly increased in patients with worst glycemic control (HbA1c≥9%) than in patients with poor control (7.0-9.0%) as well as those with excellent control (≤7%) and NLR emerged as an independent predictor of worst diabetes control. ^39^ A study reported that NLR was significantly higher in diabetic patients, patients with HOMA-IR (Homeostatic Model Assessment for Insulin Resistance) > 2, and concluded that NLR could be an effective risk predictor of insulin resistance. The study further reported that with every one unit increase in NLR, the odds of insulin resistance increased by approximately 7.2. ^40^ Another study reported that NLR was observed to be significantly increased in prediabetics, newly diagnosed diabetics, as well as chronic diabetic patients when compared to healthy individuals (manifesting normal glucose tolerance). ^41^

A study on diabetic retinopathy reported that NLR was significantly higher in patients with diabetic retinopathy. ^42^ Another study reported that NLR did not increase significantly with increasing extent of glucose intolerance, however, NLR was increased in patients with secondary complications of diabetes like neuropathy and glaucoma. ^43^ A study reported that NLR was statistically higher in diabetics with poor glycaemic control (HbA1c >7.5%) when compared to diabetics with good glycemic control group (HbA1c ≤7.5%). ^44^ Another study also reported that NLR was significantly higher in diabetic with very poor glycemic control (HbA1c>9%), when compared to patients with poor/ moderately poor control (7-9%) and patients with good glycemic control (≤7%). ^45^ Another study reported a significantly higher NLR (median 2.44) in T2DM patients when compared to control and also reported that NLR strongly correlated with fasting blood glucose and HbA1c levels. ^46^ A prospective 6-year follow-up study reported that participants with higher NLR (highest quintile) had increased odds of developing T2DM when compared to participants with low NLR. ^47^ A study reported that neutrophils were significantly higher, and lymphocytes were significantly decreased in patients with diabetic peripheral neuropathy, thus concluding that increased NLR could be an independent risk factor for diabetic peripheral neuropathy (NLR above 2.13 showed a specificity of 48.1%, sensitivity of 81.3%). ^48^

## 3. Coronary artery disease (CAD)

Formerly regarded as an exclusive lipid associated inflammatory metabolic disorder, recent advances in cardiovascular biology have highlighted the central and pivotal role of chronic inflammation, and the innate and adaptive immune systems (mediators of inflammation), in the initiation, progression, prediction, and prognostication of coronary artery diseases. ^48^^–^^51^

### 3. A) Neutrophils in Coronary Artery Disease

Neutrophils are being considered as drivers of sterile and chronic inflammatory pathologies such as atherosclerotic plaque formation. ^52^^,^^53^

**Figure 2.**
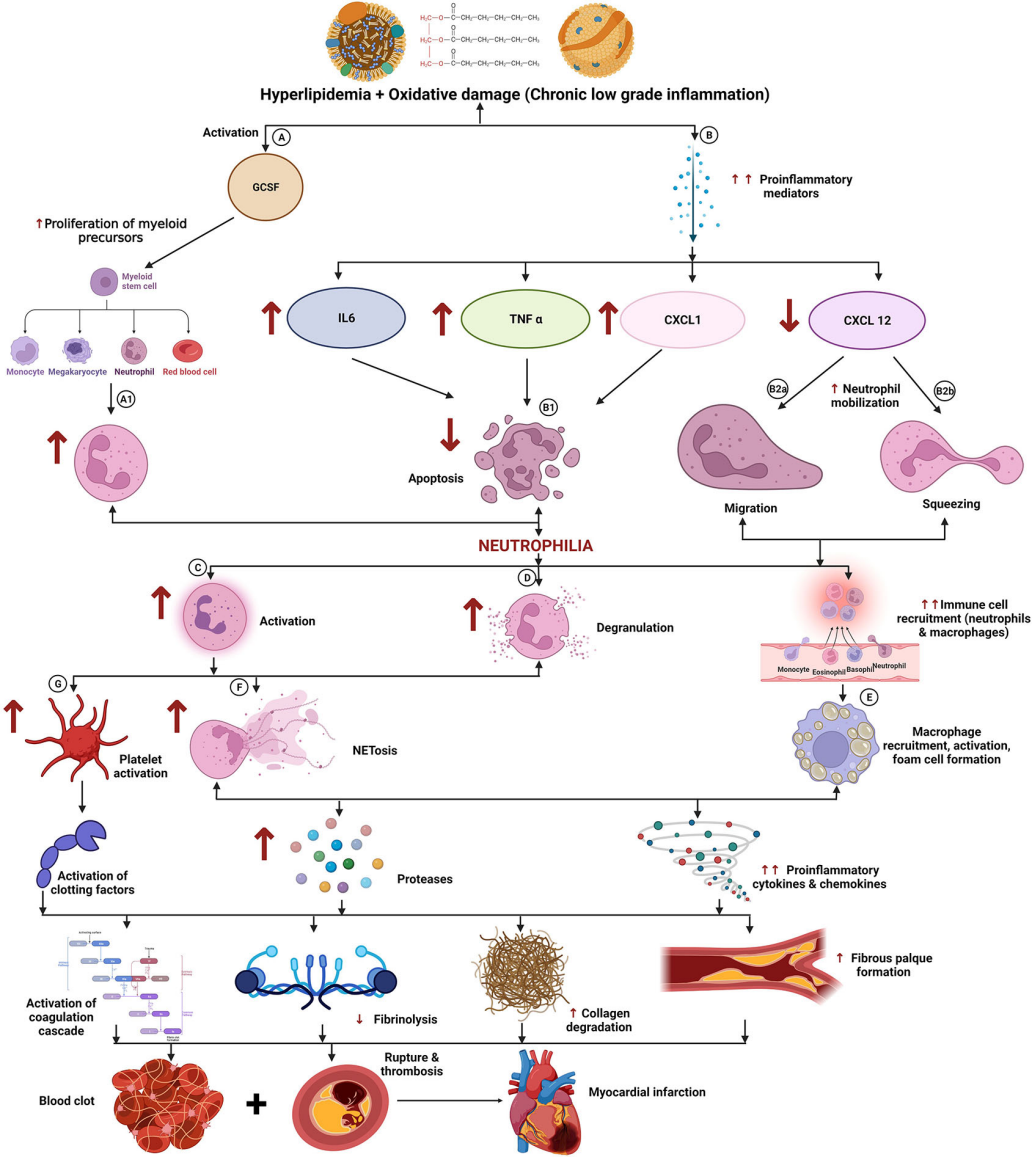
Role of neutrophils in coronary artery disease (CAD). Hyperlipidaemia and oxidative damage create a chronic low-grade inflammatory microenvironment that activates the release of granulocyte colony-stimulating factor (GCSF) (A) and pro-inflammatory mediators (B) (increase in IL6, TNF alpha, CXCL1 and decrease in CXCL 12). GCSF triggers the proliferation of neutrophils (A1), while IL6, TNF alpha, and CXCL1 retards neutrophil apoptosis (B1) which leads to an increase in neutrophil count followed by increased neutrophil activation (C) and degranulation (D). These in turn lead to the heightened NET formation (F) and platelet activation (G). Decreased CXCL12 triggering enhanced neutrophil mobilization (B2 a,b) along with activation of macrophage cascade accelerates foam cell formation (E). NETosis and foam cell formation lead to increased secretion of pro-inflammatory proteases, cytokines, and chemokines. The entire sequence of events finally culminates in plaque rupture, and thrombosis which leads to myocardial infarction (MI). Note: ↑ indicates increased levels while ↓ refers to decreased levels/concentrations. This figure is an original figure produced by the authors for this review article and has been filed for copyright with the Copyright Office, Government of India.

The various neutrophil mediated mechanisms of atherosclerotic plaque progression are:

**3.A.i. Increased neutrophil count (** **Figure 2** **):** Numerous studies have explored the alterations in neutrophil counts in CAD and have reemphasized the presence of neutrophilia in acute coronary syndromes. Neutrophil counts have been shown to correlate with the severity of atherosclerosis, infarct size, arterial stiffness, and in-hospital as well as long-term outcomes. ^54^

A prospective cohort study on 398 patients with peripheral arterial disease reported that patients showing higher tertiles (>5.8) of total neutrophil counts at admission, were at an increased risk of developing MACE (major adverse cardiovascular events), death, and composite adverse events when compared to patients in the lowest tertile. Thus, neutrophil counts add prognostic value to traditional atherothrombotic risk factors and markers of inflammation. ^55^ A community-based study on recruiting incident MI (myocardial infarction) cases reported that the overall three year survival and heart failure free survival could be predicted by neutrophil count at presentation with patients at the middle tertile showing 1.44 HR (hazards ratio) for death and 1.32 HR for heart failure when compared to those in lowest tertile, while patients in the highest tertile showing 2.6 HR for death and 2.12 HR for heart failure, the study concluded that NLR significantly improved risk stratification over traditional risk predictors. ^56^ A review of 21 studies including approximately 34,000 patients reported that neutrophil count was an independent predictor of cardiovascular outcomes. ^57^ A study on 486 suspected CAD patients undergoing coronary angiography reported that band neutrophil counts in these participants positively correlated to atherosclerosis. ^58^

Hyperlipidaemia, a major predisposing factor in atherosclerosis, promotes lipid deposition, plaque formation, vasculature damage, and is also implicated in the induction of neutrophilia. ^59^ Elevated circulating cholesterol induces granulocyte colony-stimulating factor (stimulating proliferation of myeloid precursors), reduces CXCL 12 concentration (reducing PMNs [polymorphonuclear neutrophils] clearance), and increases the circulating concentration of CXCL1, which promotes neutrophil mobilization. ^60^^,^^61^ Further, the study reported that the degree of hypercholesterolemia-induced neutrophilia positively correlated with the lesion severity while experimental mice with neutropenia showed reduced plaque sizes. ^62^ Neutrophilia seems to be associated with the maturation stage of these cells, exhibiting nuclear segmentation as well as acting as a stimulator of chemokines and cytokines. ^62^^–^^64^ Neutrophils are also believed to promote monocyte and macrophage recruitment inducing foam cell formation and thereby accelerating vascular plug formation via excess neutrophil activation secondary to endothelial cell dysfunction. ^65^

**3.A.ii. Neutrophil activation (** **Figure 2** **):** Coronary atherosclerotic lesion provokes neutrophil activation. Numerous clinical and *in-vitro* studies have investigated the role of neutrophil activation in CAD and have reported evidence of exaggerated systemic and local activation of neutrophils in acute coronary syndromes. ^66^ Activated neutrophils infiltrate and aggregate at lesion sites, release proteolytic enzymes (elastase, MPO [myeloperoxidase]), ROS, etc which can directly induce endothelial and vasculature damage and exert thrombotic/hemostatic effects. ^67^

**3.A.iii. Neutrophil aggregation:** Clinical investigations reported altered neutrophil function in coronary artery disease and acute coronary syndromes. A study reported significantly higher neutrophil aggregation and oxidase activity while superoxide generation was significantly decreased in the coronary sinus of patients with CAD than in aorta thus highlighting the potential role of granulocytes in neutrophil-endothelial interactions and tissue damage. ^68^

**3.A.iv. Neutrophil extracellular traps and NETOSIS (** **Figure 2** **):** Neutrophils play myriad roles in acute and chronic inflammatory pathways, NETosis being one among them. As a damage response, neutrophils extrude their DNA (NETosis) content along with the secretion of inflammatory mediators. The process of NET formation involves multiple signals triggering several receptors, molecules, and enzymes. NETosis, a special form of programmed cell death, proceeds either through “suicidal/lytic” (via the action of NADPH oxidase) or “vital” (vesicular chromatin expulsion followed by generation of anuclear neutrophil) pathways depending upon the stimulating events. ^69^ The lytic pathway proceeds through the generation of ROS, activation of protein-arginine deiminase 4 (PADA 4), and subsequent chromatin decondensation via the action of secretory enzymes like MPO and NE (neutrophil elastase) and histone citrullination. The vital pathway on the other hand is initiated through pattern recognition molecules in response to bacterial LPS and activated platelets and proceeds via chromatin blebbing and exocytosis generating an anuclear neutrophil that gets destined to undergo programmed cell death. The released NETs contain negatively charged extruded decondensed chromatin material which forms the backbone, along with histones, nuclear and cellular proteins, cytoskeleton, proteases, and tissue factors that organise into networks that can physically entrap microbes and other circulating elements in the blood. ^69^^,^^70^ NETosis has been known to play a pivotal role in clearance of microbes and a strong pillar of the human innate immune response.

Chronic inflammation, persistent stimulation or an exaggerated response to injury likely triggers excess NETosis which has been strongly implicated in atherosclerosis progression and thrombosis. Circulating cell free double strand DNA is recognized as a “danger-associated molecular pattern” (DAMP) and can induce an exaggerated activation of innate immunity, whereby inflammation-induced DNA damage and DNA-induced inflammation form a vicious cycle. A study on 111 STEMI patients undergoing PCI (percutaneous coronary intervention) and thrombectomies reported that NET burden in thrombus positively correlated with infarct size and negatively correlated with ST segment resolution and vice versa with lesion site DNAse activity. ^71^ Another study on 37 ACS (acute coronary syndromes), and 58 AIS (acute ischemic stroke) patients reported significantly higher concentrations of dsDNA when compared to the control group, thus circulating level of NETs was increased in patients with ACS and AIS at initial presentation. ^72^ A study on paraffin-embedded coronary plaque segments reported an abundance of neutrophils and NETs in complicated plaques (IPHs, erosions, ruptures) when compared to intact ones thus concluding that NETosis is a prominent prothrombotic participant which facilitates the progression of thrombotic or haemorrhagic complications in coronary artery disease leading to precipitation of acute coronary syndromes. ^73^

**3.A.iv.a) NETs in Atherosclerosis:** The exact role of NETosis in atherosclerosis remains to be elucidated. However, based on available evidence, it is believed that cholesterol crystals might stimulate NETosis and trigger a vicious cascade resulting in enhanced secretion of inflammatory cytokines, inflammasome activation, priming of macrophages, activation of T _H_17 helper cells, and neutrophil recruitment at the lesion site. NETs are also implicated in the oxidation of HDL (high density lipoprotein), thereby reducing their functional cholesterol efflux capacity, endothelial cell activation, dysfunction and apoptosis (direct vasculature damage), and generation of anti-double-stranded-DNA autoantibodies. ^74^

**3.A.iv.b) NETs in Atherothrombosis:** NETs are believed to exert strong thrombotic effects. Impaired collagen synthesis and overexpression of collagenases by inflammatory mediators disrupt the fibrous cap leading to plaque erosion, these lesions house high amounts of NETs which can stimulate the coagulation cascade. This would result in thrombus generation and occlusion or blockage of arteries. ^69^ Multiple studies have reported the presence of copious amounts of neutrophils and NETs in coronary thrombi isolated from ACS patients. A study on 26 MI patients undergoing thrombectomies provided evidence of interactions between activated platelets and neutrophils, where activated platelets stimulate NET formation. ^75^ Evidence postulates a vicious cycle of chronic inflammation where activated neutrophils further activate more platelets via proteins expressed and embedded in the NETs. NETs bind coagulatory proteins like von Willebrand factor and fibronectin (promoting platelet binding), coagulation factor XII (generating FIa, a clot stabiliser, and platelet activator thrombin), and fibrinogen. ^54^

NETs thus accelerate thrombin generation through multiple pathways including platelet activation and blocking protein C thrombomodulin action. Further, NETs have also been shown to increase thickness and decrease the permeability of fibrin in blood clots and interfere with tPA (tissue plasminogen activator) directed fibrinolysis. NETs are the indispensable vital weapons of our innate immunity with strong microbicidal activity, however, hazards of chronic activation of NETs via sterile inflammatory pathways lead to disastrous complications. ^70^

Hence the scope of targeting and downregulating inflammatory cascades is being explored as a newer and auxiliary therapeutic target in atherosclerosis. Targeting a robust arm of our immunity like the neutrophils is extremely delicate and carries its own perils (disarming the host’s ability to fight acute infections). However, selectively targeting of the key mediators implicated in atherosclerosis and atherothrombosis, is definitely worth exploration and numerous clinical trials are underway to explore the benefit to risk ratio of such anti-inflammatory therapies targeting neutrophils and NETosis pathways (CANTOS trial, use of DNase 1). ^70^

Further research is underway to determine the key differences in pathogenic versus sterile inflammatory neutrophil activation, insights into which may pave possibilities for selective inactivation/inhibition of neutrophil activation through non-infectious chronic inflammatory pathways.

**3.A.v. Phenotypic modifications in circulating neutrophils:** Investigations are being carried out to explore phenotypic alterations in neutrophils and its association with coronary artery disease. A study reported increased band neutrophils and total MPO in STEMI patients while NSTEMI patients manifested higher neutrophil side scatter signal intensity. ^72^ Band cells represent immature neutrophils released to the circulation by some inflammatory stimulus to satiate the high demand for neutrophils probably as a response to severe myocardial damage. The study further reported that the percentage of low-density neutrophils correlates with NLR. ^72^

**3.A.vi. Delayed neutrophil apoptosis (** **Figure 2** **):** The neutrophils undergo apoptosis, a regulated programmed cell death, to aid in terminating the inflammatory cascade and bring about resolution of inflammation. However, delayed neutrophil apoptosis has been implicated in CAD and ACS. A study reported delayed PMN apoptosis in ACS patients. ^76^ Another study reported reactivation of telomerase activity in neutrophils isolated from plaques of ACS patients. ^77^ Another study reported delayed apoptosis of circulating peripheral neutrophils in unstable angina patients. The study also reported that hs CRP (high sensitive C reactive protein) levels correlate with the delay in neutrophil apoptosis in these patients indicating that inflammatory cytokines might exert effects on neutrophil survival. ^78^ Several studies on IL6 (interleukin 6), GCSF (granulocyte colony stimulating factor), interferon gamma, and TNF alpha have shown to reduce apoptosis in neutrophils. Thus, multiple PMNs survival/anti apoptotic pathways operate at peripheral and local levels and prolonged survival of neutrophils can translate into higher inflammatory activities at the vulnerable plaque microenvironment triggering a vicious cascade of inflammation that culminates into an acute coronary event. ^78^

### 3. B) Lymphocytes in CAD

Recent advances in cardiovascular biology have brought to light the role of immune responses and lymphocytes in atherosclerosis. It is now believed that lymphocyte mediated immune responses against plaque (modified self-antigens) antigens are associated with extensive inflammation and progression of plaque development. ^79^

**3.B.i. T lymphocytes:** The T lymphocytes play a crucial role in immune homeostasis by modulating their role through the entire spectrum of immune stimulation to immune suppression. Lymphocyte progenitors migrate to the thymus to generate mature TCR alpha/beta CD4 ^+^ and CD8 ^+^ T cells, and TCR gamma/delta T cells. ^80^ Studies report that majority of the T cells isolated from human atherosclerotic plaques display an effector memory phenotype (including CD4 ^+^ T cells and Th 1 subtype). ^81^^,^^82^ TCR activation via stimulation by antigens and cytokines, results in the differentiation of naive CD4 T cells into distinct Th cell lineages, such as Th1 (proinflammatory) cells secreting interferon gamma, Th2 (anti-inflammatory) cells secreting IL 4, IL 5, and IL 13, Th17 (proinflammatory) cells secreting IL 17 and IL 22, and iTreg cells, the immunosuppressive arm of the Th cell lineage. ^83^ CD8 ^+^ cytotoxic T cells are also known to be abundant in human atherosclerotic plaques. ^84^

**Figure 3.**
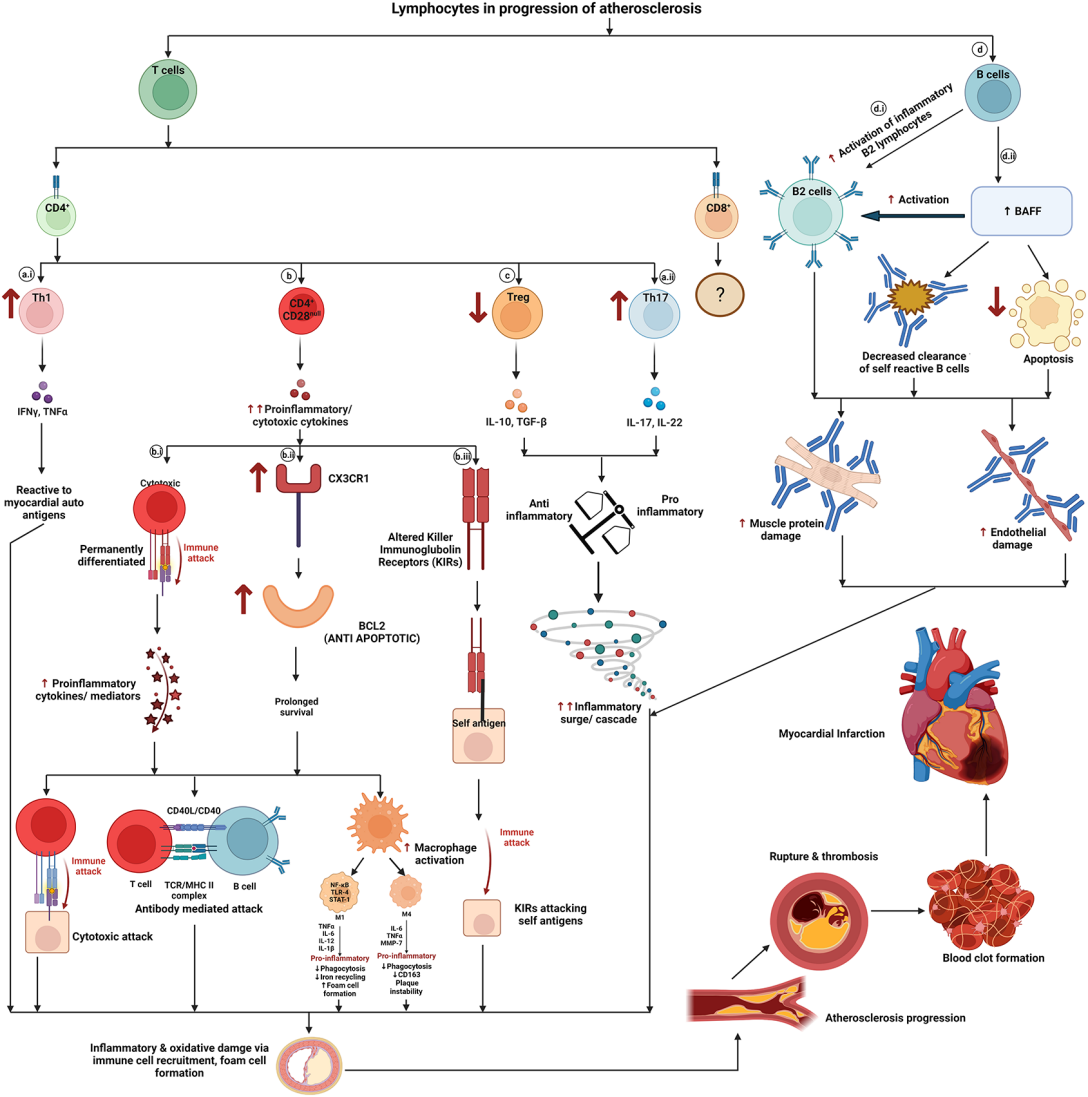
Role of lymphocytes in coronary artery disease (CAD). CD4 ^+^ T lymphocytes play a vital role in coronary artery disease. Hyperlipidemia and oxidative damage create a chronic low-grade inflammatory microenvironment which stimulates the increased differentiation of CD4 ^+^ T cells to Th1 (a.i) and Th17 (a.ii) phenotypes and decreased production of the regulatory Treg cells (c). Further, chronic inflammation triggers the generation of unusual CD4 ^+^CD28 ^NULL^ T cells (b). b.i) These are permanently differentiated cytotoxic cells. b.ii) These cells have heightened expression of CX3CR1 which results in increased expression of the anti-apoptotic BCL2 protein. b.iii) These cells express altered Killer Immunoglobulin Receptors (KIRs) which show increased reactivity to self-antigens. c) Perturbation of the Th17 and Treg cell equilibrium further exaggerates the proinflammatory microenvironment. d) B lymphocytes in CAD are shown to manifest increased differentiation of the proinflammatory B2 phenotype (d.i) along with increased expression of BAFF (d.ii). BAFF further aids the B2 cell maturation and increases their resistance to apoptosis. a+b+c+d) Increased inflammatory and oxidative damage of the endothelial membrane through immune cell recruitment accelerates foam cell generation and plaque maturation. The entire sequence of events finally culminates in thrombosis which leads to myocardial infarction (MI). Note: ↑ indicates increased levels while ↓ refers to decreased levels/concentrations. This figure is an original figure produced by the authors for this review article and has been filed for copyright with the Copyright Office, Government of India.

**3.B.i.a) CD4** ^**+**^ **T cells**:

**I. T cell activation (** **Figure 3** **):** Experimental studies have reported evidence of heightened T cell activation, specially the Th1 subtype, in atherosclerosis. and reactivity to myocardial autoantigens. ^81^^,^^82^

A study reported CD4 ^+^ T cell activation and proliferation in mouse MI models. ^81^^,^^82^

**II. Unusual CD4** ^**+**^ **CD28** ^**null**^ **T cells (when helpers become killers) (** **Figure 3** **):** CD4 ^+^CD28 ^null^ cells, an unusual subset of CD4 ^+^ T helper cells, have been implicated in atherosclerosis and poor prognosis of CADs. ^85^ In absence of a CD28 mediated signal, T cells remain inactive, and their proliferation is inhibited. However, in absence of CD28 membrane receptor, these unusual CD4 ^+^CD28 ^null^ T lymphocytes lose the signal mediated immunoregulation feature and are permanently differentiated expressing cytotoxic (functional resemblance with natural killer cells) and proinflammatory characteristics. CD4 ^+^CD28 ^null^ T cells are a unique T cell subset which have been extensively implicated in CAD due to their numerous proatherogenic characteristics. ^85^

➢ **Proinflammatory nature of CD4** ^**+**^ **CD28** ^**null**^ **cells:** CD4 ^+^CD28 ^null^ T cells represent a class of terminally differentiated cytotoxic cells which release substantially increased concentration of proinflammatory cytokines (interferon-ϒ, TNF-α, and IL) and cytolytic enzymes (perforin, granzyme A and B). The result is exaggerated T cell and macrophage recruitment, activation and differentiation of macrophages, generation of foam cells, plaque destabilization and rupture. Interferon gamma is also implicated in inhibition of collagen synthesis which might additionally contribute to plaque instability and rupture. ^85^
➢ **Stimulatory killer immunoglobulin like receptors (KIRs):** CD4 ^+^CD28 ^null^ cells express a variety of altered receptors (resembling that of natural killer cells) known as the killer immunoglobulin-like receptors (KIRs), a family of receptors which recognise class I MHC (major histocompatibility complex) molecules and are involved in self-tolerance mechanisms. ^85^ A study reported that CD4 ^+^CD28 ^null^ T cells isolated from ACS patients expressed a broader spectrum of KIRs (most abundant being CD158j, the stimulatory variant) when compared to healthy controls. The study further reported de novo expression of DAP12 (adapter chain transmitting CD158j signals which activates MAPK [mitogen activated protein kinase] cascade) in CD4 ^+^ T cells of patients, thus furnishing chronic cytolytic stimulation and competence of CD4 ^+^CD28 ^null^ T cells even in absence of exogenous antigen mediated T cell receptor stimulation. ^86^
➢ **Prolonged survival:** CD4 ^+^CD28 ^null^ cells express CX3CR1 receptor which makes the cells resistant to the usual apoptotic signals via upregulation of Bcl 2 (anti apoptotic protein). ^87^ A study reported significantly increased levels of circulating CD4 ^+^CD28 ^null^ T cells in unstable angina when compared to patients with chronic stable angina. These cells have been associated with atherosclerotic plaque instability, severity or extent of atherosclerosis, poor prognosis, and long-term MACE. ^88^^,^^89^

Further, CD4 ^+^CD28 ^null^ T expansion in atherosclerosis has been found to be oligoclonal in nature, implicating chronic antigen stimulation by common antigens (most probably heat shock proteins), and these subsets have been found to preferentially accumulate in ruptured plaques thus implying their role in plaque destabilization. ^85^^,^^90^

**III) Low and dysfunctional Tregs (** **Figure 3** **):** Tregs, the crucial immunosuppressive arm, regulates both innate and adaptive immune responses by effectively inhibiting the production of proinflammatory cytokines, thus playing an indispensable role in immune homeostasis. Numerous studies have emphasized the role of Treg cells in acute coronary syndromes. Studies have reported lower levels of circulating as well as intra lesional (local atherosclerotic lesions) Tregs in ACS patients when compared to patients with stable angina as wells as in healthy controls. ^91^^,^^92^ A study reported that low levels of baseline circulating CD4 ^+^ Foxp3 ^+^ T cells is associated with a higher risk of future MI. ^93^ The possible causes of the reduction in circulating Tregs number during MI include impaired output from thymus, increased apoptosis, and trafficking of peripheral Tregs to inflammatory sites. Further, a study on an animal model has reported that adoptive transfer of Treg cells could improve left ventricular contraction after MI, effectively negate MI-induced left ventricular remodelling, downregulate interferon gamma expression in hearts and upregulate splenic Foxp3 expression. ^94^

**IV) Th17/Treg imbalance (** **Figure 3** **):** The imbalance between anti-inflammatory Treg cells (secreting transforming growth factor beta and IL 10) and pro inflammatory Th17 cells (producing IL 17) with a Th17 favouring trend is implicated in pathogenesis of coronary artery disease and acute coronary syndrome. ^95^ Studies have reported significantly elevated Th17 cells, decreased Treg counts and function and decreased Treg/Th17 ratio in ACS patients. Another study reported baseline Th17/Treg ratio (at admission) to be a predictor of mortality in MI patients with cut-off of >0.33 to be an independent and a sensitive predictor of 28 days as well as one year mortality in these patients. ^95^^,^^96^

**3.B.i.b) CD8** ^**+**^ **T cells:**

The role of CD8 ^+^ cells in atherosclerosis is yet to be explored in detail and existing evidence are contrasting on whether they serve as proatherogenic or exert an atheroprotective effect. ^97^^,^^98^

**3.B.ii) B lymphocytes:**

B lymphocytes play prime roles in innate and adaptive immune responses. Their role in infectious diseases is well characterised, however the recent evidence highlights their involvement in chronic inflammatory diseases. A recent network-driven integrative analysis of data from genome wide association studies and whole blood gene expression profiles from Framingham Heart Study participants identified B cell immune responses as causative for CAD. ^99^^,^^100^ The B lymphocytes can differentiate into mature B2 subset which develops through Th dependent delayed type response and results in the formation of high affinity antibodies. These antibodies are very potent mediators of immune response, B1 subset (minor), non-circulating B cells, which secrete nonspecific IgM class of polyreactive antibodies that constitute the first line of defence. Literature evidence imply B2 lymphocytes to be proatherogenic while the minor B1 subset to be exerting atheroprotective effects. ^101^

**3.B.ii.a) Preferential activation of B2 lymphocytes (** **Figure 3** **):** While only a few B lymphocytes locally accumulate within the atherosclerotic lesions, a substantial number are found in the adventitia of the culprit vessels which implies a chronic immune activation response. Further, the oligoclonal antigen mediated proliferation of B2 cells lead to generation of high affinity antibodies, assembling apolipoprotein B 100, and transgelin. ^102^ This further steers in production of pro inflammatory cytokines by both antibody dependent and independent mechanisms which aggravate atherosclerosis.

**3.B.ii.b) Role of BAFF (** **Figure 3** **):** B cell development and survival are regulated by various factors, the B cell activating factor (BAFF), a member of the tumour necrosis factor family, being one of the most important. BAFF is indispensable for B cell (especially the proatherogenic B2 subset) maturation and also aids the survival of low-affinity self-reactive B lymphocytes. ^101^^,^^103^ Evidence reiterates that high circulating concentrations of BAFF and CCL7 in MI patients is associated with increased risk of mortality and recurrent infarction. ^101^ An experimental study reported that deficiency of BAFF receptors resulted in significantly decreased levels of B2 cells but did not alter the number or function of the B1a (atheroprotective) subtype. ^103^

It was further reported that BAFF receptor-deficient models showed diminished progression of the atherosclerotic lesion and reduced lesion size. B cell depletion via injection of CD20 monoclonal antibody during the golden/acute phase of MI has been reported to markedly improve cardiac function and reduce infarct size. ^103^

### 3. C) NLR in CAD

NLR, a novel systemic inflammation hematological index, is being widely explored in coronary artery diseases and its short- and long-term outcomes. ^57^^,^^104^ NLR has been reported to be an independent predictor of MACE and mortality in STEMI. NLR could also be a predictor of critical stenosis and is associated with both the severity and plaque morphology of coronary atherosclerotic disease. ^105^ In a study analyzing the baseline and post treatment NLR of patients from five randomized clinical trials, it was reported that baseline NLR was a reliable predictor of incident events and mortality and every quartile increase in NLR increased the risk of MACE by 9% to 31% in different RCTs. ^106^ Another study reported NLR with a cut off of 2.59 could be a reliable and independent predictor of high residual SYNTAX score in STEMI patients undergoing PCI. ^107^ A high NLR emerged to be an independent prognostic indicator for survival in both CAD and T2DM patients with NLR ≤ 2.5 showing significantly favorable overall survival. ^108^ A study reported that elevated NLR is associated with larger infarct, future MACE/poor prognosis, and abnormal cardiac remodeling. ^109^ A follow-up study assessing cardiovascular death in 181 STEMI patients undergoing emergency PCI reported statistically significant differences in NLR, CRP, NT pro BNP (N terminal pro brain type natriuretic peptide) in the mortality group when compared to surviving patients ^110^

A study on 111 NSTEMI patients with slow flow coronaries reported that the incidence of recurrent angina and MI was significantly higher in highest NLR tertile patients and NLR could be an independent predictor of recurrent MI. ^111^ Another study on 744 STEMI patients reported high NLR was associated with a statistically significant higher risk of mechanical complications of STEMI (sudden cardiac arrest, stent thrombosis, pericardial effusion, post myocardial infarction (MI) pericarditis, and post MI ventricular septal rupture, free-wall rupture, left ventricular thrombus, and acute mitral regurgitation during hospitalization). ^112^

**Imbalance of the autonomic nervous system**

Neutrophils have adrenergic receptors which are regulated by the sympathetic arm, while lymphocytes bear cholinergic receptors responsive to the parasympathetic nerve endings. It is postulated that dysregulation/imbalance of the autonomic nervous system might stimulate atherosclerosis, studies reaffirm this postulation and report that increased sympathetic stimulation is in fact associated with the exaggerated synthesis of proinflammatory cytokines which contribute to vascular lesions. Since a perturbed balance between the 2 systems is being implicated in the progression of atherosclerosis, NLR might mechanistically be a reliable indicator of the balance between the two arms of the autonomic nervous system. ^16^

## 4. Chronic obstructive pulmonary disease (COPD)

COPD, an inflammatory lung pathology progressing to emphysema and chronic bronchitis, involves strong contributions of both innate and adaptive arms of the immune system and is shown to exert both local as well as systemic inflammatory effects. ^113^

### 4. A) Neutrophils in COPD

Copious evidence emphasizes the pivotal role of neutrophils in COPD, neutrophils being the predominant inflammatory cells in the bronchi and respiratory lumen of COPD patients ^113^ Advances in neutrophil biology have indicated multiple and complex neutrophil functional responses, including priming, enhanced responsiveness (degranulation & oxidative burst), response to hypoxia, cytokine, and chemokine release.

**Figure 4.**
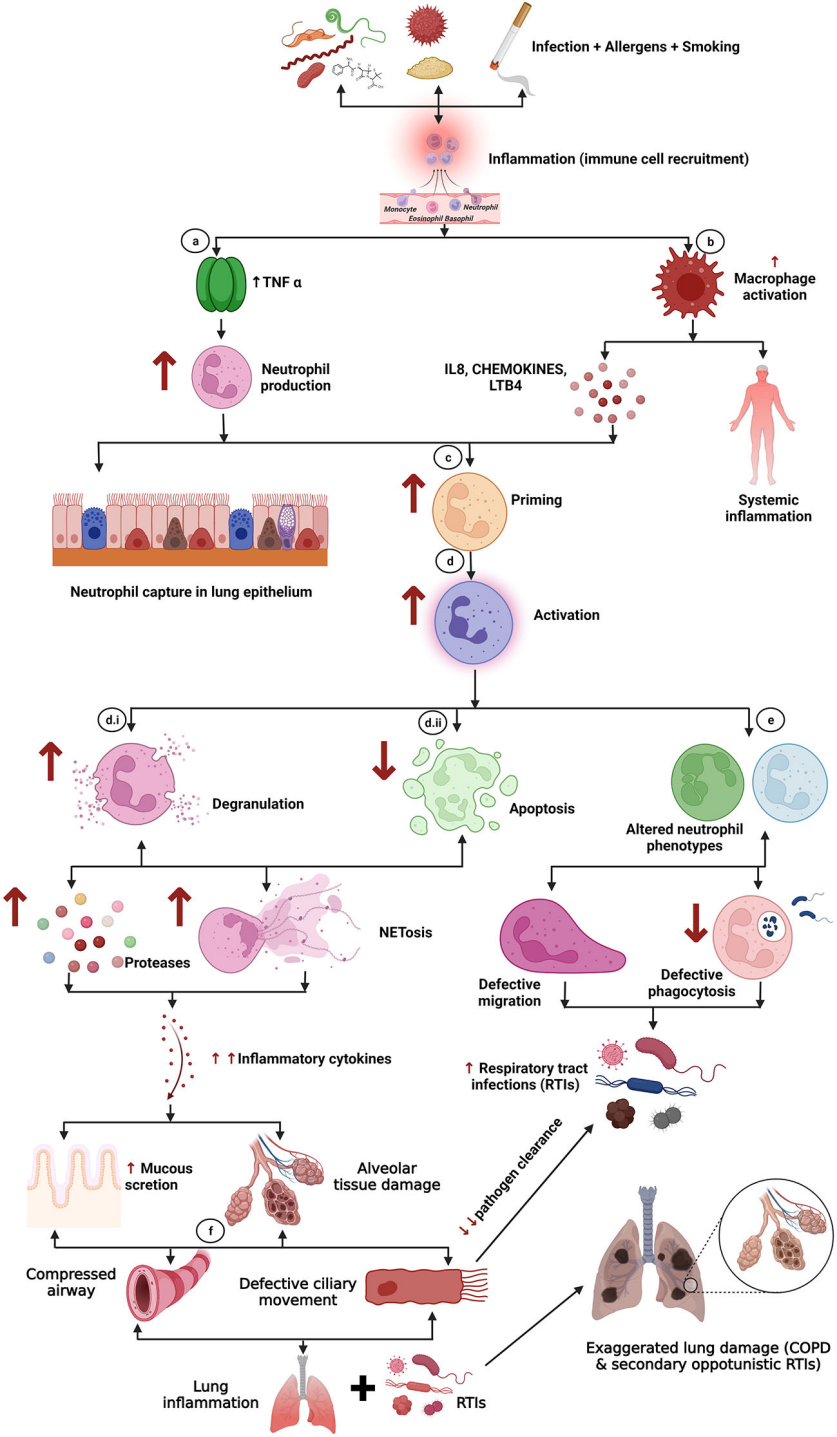
Role of neutrophils in chronic obstructive pulmonary disorder (COPD). Exposure to allergens, respiratory tract infections (RTIs), and cigarette smoking generate an inflammatory microenvironment that leads to immune cell recruitment. Increased immune cell recruitment leads to (a) increased TNF alpha secretion which stimulates neutrophil production, (b) macrophage recruitment and activation which triggers an increased release of inflammatory mediators. (c) Exaggerated release of inflammatory mediators also triggers increased neutrophil priming and results in (d) increased neutrophil activation which leads to increased neutrophil degranulation and increased resistance to apoptosis (e) along with the generation of neutrophils with altered phenotypes. These further result in compressed/collapsed airways and defective mucociliary pathogen clearance (f). e+f) Further intensifies sterile lung inflammation as well as damage due to colonization by opportunistic pathogenic microorganisms which culminates in widespread lung damage in acute exacerbations or chronic long-term COPD. Note: ↑ indicates increased levels while ↓ refers to decreased levels/concentrations. This figure is an original figure produced by the authors for this review article and has been filed for copyright with the Copyright Office, Government of India.

The major mechanism of neutrophil-mediated perturbations in COPD include:

**4.A.i. NETosis (** **Figure 4** **):** Recent studies have shown significant interest to investigate the role of NETs in COPD, but the current evidence is conflicting. Increased percentage of NET producing neutrophils as well as elevated quantities of sputum NET components have been reported in both stable and exacerbating COPD. Further, sputum NET concentrations have been shown to correlate with the severity of airflow limitation. Increased NET generation in COPD can lead to rapid progression of pulmonary damage by triggering damage and death of endothelial and epithelial cells. ^114^ However, circulating neutrophils from patients experiencing COPD exacerbations have been reported to manifest a diminished ability to undergo NETosis despite the increased presence of cell-free DNA in plasma. A study reported decreased NET formation and increased cell-free DNA in patients experiencing acute exacerbations when compared to stable patients. These authors concluded that while lower NET production inhibits bacterial clearance (increasing susceptibility to respiratory tract infections), elevated cell free DNA concentrations might add to systemic host damage by curtailing inflammatory resolution. ^115^

**4.A.ii. Neutrophil dysfunction (** **Figure 4** **):** Neutrophils in COPD patients have been reported to exhibit numerous functional perturbations. It is reported that chronically activated neutrophils in COPD patients show decreased phagocytic abilities. A study reported significantly lower phagocytic and lytic indices in neutrophils of COPD patients when compared to healthy controls. The mechanism behind this impairment remains to be clearly understood, however, it is hypothesized that this phagocytic dysfunction could be related to neutrophilic protease-mediated cleavage of opsonins and their receptors, neutrophil elastase mediated C3bi and CR1 cleavage (compromising complement-mediated phagocytosis) or downregulation of Toll-like receptor 2 expressions.

A study reported hypoxia-induced impairment of bactericidal activity in neutrophils of COPD patients thus making the patients susceptible to opportunistic respiratory tract infections. Further, another study postulated the presence of intrinsically defective neutrophils in COPD because circulating neutrophils from COPD patients showed increased speed but decreased chemotactic accuracy irrespective of the GOLD (Global Initiative for Chronic Obstructive Lung Disease) Stage. Further hypoxia-induced oxygen tension has also been implicated in prolonging neutrophil survival and perhaps inducing proinflammatory alterations in neutrophil function (enhanced degranulation and proinflammatory protease secretion, dysfunctional phagocytosis). ^116^^,^^117^

**4.A.iii. Enhanced neutrophil priming (** **Figure 4** **):** Neutrophil priming is one of the most potent proinflammatory perturbation being explored in chronic diseases including COPD. A study on circulating neutrophils of COPD patients reported enhanced respiratory burst and neutrophil response to proinflammatory stimulation. ^118^ Another study reported increased CD11b and decreased CD62L expression in circulating as well as airway neutrophils of chronic smokers with and without COPD which indicates a higher state of priming. ^119^

**4.A.iv. Neutrophilia (** **Figure 4** **):** Airway neutrophilia is a characteristic feature of inflammatory lung pathologies and their progression. A study reported elevated neutrophil count to be an indicator of poor prognosis and mortality in COPD. ^120^ Another study reported that the percentage of neutrophils in the sputum of COPD patients showed a progressive increase with an increase in the GOLD stage and was weakly associated with FEV _1_ (forced expiratory volume in 1 second) %. ^121^

**4.A. v. Delayed neutrophil apoptosis (** **Figure 4** **):** A study reported significantly lower pro-apoptotic Bak and higher anti-apoptotic Bcl-xl and Mcl-1 expression in COPD patients. ^122^ Physiologically, apoptosis of neutrophils ensures the prevention of widespread tissue damage as degranulation is checked and the apoptotic neutrophils are cleared from circulation without spillage of inflammatory neutrophilic contents. Proinflammatory stimulations such as lipopolysaccharide, GM-CSF (granulocyte macrophage colony stimulating factor), hypoxia as well as nicotine inhibit neutrophil apoptosis which in turn triggers a vicious cascade of chronic inflammation. Further, studies have reported higher surface expression of Mac-1 and lower expression of L-selectin in apoptotic neutrophils of COPD patients which is indicative of an exaggerated activation, such activated neutrophils are potent to initiate and propagate tissue damage even during apoptosis. ^123^

**4.A.vi. Neutrophil migration (** **Figure 4** **):** A study reported that neutrophils of smokers (with/without COPD) showed exaggerated chemotactic response to CXCL8. ^124^ Another study reported variations in migratory kinetics, accuracy as well as structural adaptations in neutrophils of COPD patients, the study reported that neutrophils derived from COPD patients had a greater speed of migration but manifested diminished accuracy and formed fewer migratory pseudopods. ^125^ Another study reported that treatment with sputum from COPD patients triggered neutrophil chemotaxis in a concentration-dependent manner while pre-treatment of sputum or neutrophils with anti–IL-8 antibody or the LTB _4_ (leukotriene B4) antagonist, demonstrated a concentration dependent inhibition of sputum induced neutrophil chemotaxis, which opens up new therapeutic targets in the management of COPD. ^126^

**4.A.vii. Altered neutrophil proteinases:** Neutrophil proteinases are hypothesized/reported to be abnormal in COPD. Neutrophil elastase, a major inflammatory neutrophil proteinase, derived from neutrophils of COPD patients is reported to exhibit resistance to AAT (alpha 1 anti trypsin) inhibition and retain ECM (extracellular matrix) binding as well as ECM protein degrading properties which might greatly accelerate lung parenchyma damage. ^127^ COPD neutrophils also exhibit exaggerated release of histotoxic neutrophil proteases which lead to widespread tissue injury. Further P-gp, a neutrophilic chemoattract derived from MMP (matrix mettaloproteinases) induced enzymatic degradation of collagen, is also reported to be elevated in the blood and sputum of COPD patients. ^117^ Serine proteinases are potent stimulators of mucus secretion which reduces mucociliary clearance, contributes to airway obstruction, and increases susceptibility to bacterial colonisation. ^127^

**4.A.viii. Altered Neutrophil phenotypes (** **Figure 4** **):** Subtly altered neutrophil phenotypes have been implicated in COPD. A study found no alteration in neutrophil activation markers (CD11b, CD66b, CD62L) but reported a downregulated expression of surface chemokine receptors for CXCL8, CXCR2. ^128^ Another study also reported unaltered neutrophil activation markers CD11b and CD66b and subtle reductions in the surface expression of CXCR2 (a chemokine receptor), CD10 (a common maturity marker) and CD62L (an adhesion molecule) in neutrophils from patients with COPD. ^129^

### 4. B) Lymphocytes in COPD

COPD, a chronic inflammatory lung pathology, has been associated with altered lymphocyte count and function.

The major mechanism of lymphocyte-mediated perturbations in COPD include:

**Lymphocytopenia:**

Lymphocytopenia has been strongly implicated as a marker of multiple chronic inflammatory pathologies and has been shown as a marker/prognostic indicator of disease severity Studies have reported low lymphocyte count in COPD to be associated with lung function, exercise tolerance, quality of life, disease severity, prognosis, etc. ^130^ Evidence reports enhanced apoptosis of both CD4 ^+^ and CD8 ^+^ subtypes, though apoptosis of CD4 ^+^ cells seems to predominate in stable COPD. ^131^ Chronic activation and the resultant enhanced apoptosis could set up an amplified inflammatory cascade triggering progressive lung damage. ^132^ Studies have reported enhanced T lymphocyte apoptosis in airways of COPD patients which could result in secondary necrosis, retention of apoptotic material, and perpetuation of the inflammation in COPD. ^131^^,^^133^^,^^134^

Multiple mechanisms of lymphocytopenia have been proposed which include chronic activation of lymphocytes and resultant increased lymphocyte apoptosis, stress-induced lymphocytopenia (due to enhanced production of cortisol and pro-inflammatory cytokines), and inflammation-induced tissue trafficking/redistribution of lymphocytes to the site/tissue of damage. ^130^^,^^135^^,^^136^

**4.B.i) Role of T lymphocytes (** **Figure 6** **):**

**4.B.i.I) CD8+ T lymphocytes:**

**a) Activation of CD8+ T cells (** **Figure 6** **):** Studies emphasize that COPD is characterized by a chronically activated state of CD8 ^+^ T lymphocytes.

T lymphocytes undergo a sequential activation characterized by the expression of specific surface receptors that are CD45RA in naive cells, CD45R0 in mature cells, and finally CD45RA in effector/cytotoxic T lymphocytes. ^132^ A study reported a significantly higher proportion of activated CD8 ^+^ T lymphocytes (expressing CD45RA and perforin) in COPD patients (than healthy smokers) which could perpetuate extensive inflammation of tissue injury. ^137^ COPD is characterized by increased apoptosis of lung parenchyma whereas normal lung houses almost negligible apoptotic cells. ^131^^,^^138^ This widespread cell damage in lung parenchyma of COPD is probably due to the increased number, activation, and tissue redistribution of cytotoxic CD8 ^+^ T lymphocytes which mediate tissue damage and necrosis through exaggerated production of inflammatory cytokines, granzymes, and perforins.

**b) Increased numbers of CD8** ^**+**^ **CD28** ^**null**^ **T cells (** **Figure 6** **):** CD8 ^+^CD28 ^null^ cells, an unusual subset of CD8 ^+^ T helper cells (oligoclonal expansion of which has been reported in conditions leading to chronic activation of the immune system), lack CD28 membrane receptor (involved in B7 mediated signal transduction) involved in activation of T cell-mediated inflammatory immune response. The absence of CD28 membrane receptor renders these unusual CD8 ^+^CD28 ^null^ T lymphocytes to lose their signal mediated immune activation/anergy feature and destines these cells as permanently differentiated cytotoxic cells exhibiting intense and unchecked proinflammatory signals (cytokines and granzymes’). These cells are functionally active and produce increased levels of the proinflammatory cytokine IFN-γ, and the cytotoxic mediators’ granzyme b and perforin. ^133^

Studies have reported increased serum as well as tissue (local) concentrations of CD8/CD28 ^null^ T cells in COPD patients (with a history of current or past smoking), further, these cells were shown to be higher among healthy smokers when compared to healthy non-smokers. ^139^^,^^140^

**Figure 5.**
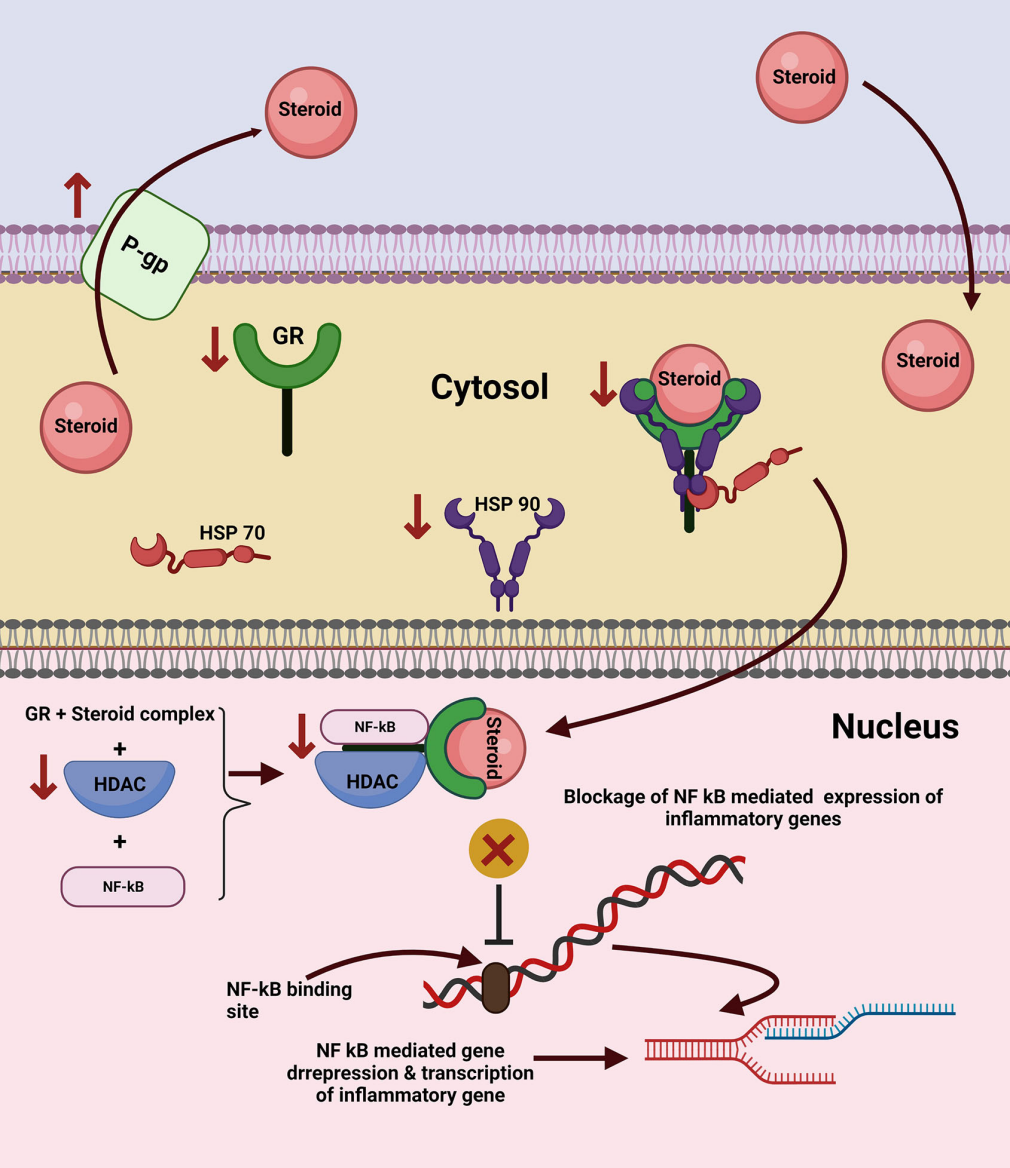
Mechanism of steroid resistance in COPD. In normal circumstances, after the transport of steroids in the cytoplasm, the steroid molecule forms a complex with the glucocorticoid receptor and heat shock proteins, HSP 70 and HSP 90. This complex is then translocated through the nuclear member where the Glucocorticoid receptor (GR)-Steroid complex is released. The released complex interacts with histone deacetylases (HDAC) and NF kB (Nuclear Factor kappa-light-chain-enhancer of activated B cells), forming another complex thus preventing the binding of NF kB to the promoter region and derepression of translation of inflammatory genes. In numerous inflammatory conditions like COPD as well as a few cancers, where steroids are one of the prime treatment modalities, a significant number of patients are observed to exhibit resistance to corticosteroid treatment. One of the major reasons for steroid resistance is the increased expression of P-gp (P glycoprotein). The other mechanisms include decreased expression of the glucocorticoid receptors, HSP 90, and HDAC. These perturbations limit the formation of the receptor steroid complex, transport of the complex to the nucleus, and the binding of the complex to NF kB. Thus, NF kB freely binds with the promoter region of the gene which undergoes derepression triggering the expression of inflammatory genes thereby rendering decreased efficacy of the prescribed steroid therapy. Note: ↑ indicates increased levels while ↓ refers to decreased levels/concentration. This figure is an original figure produced by the authors for this review article and has been filed for copyright with the Copyright Office, Government of India.

**Figure 6.**
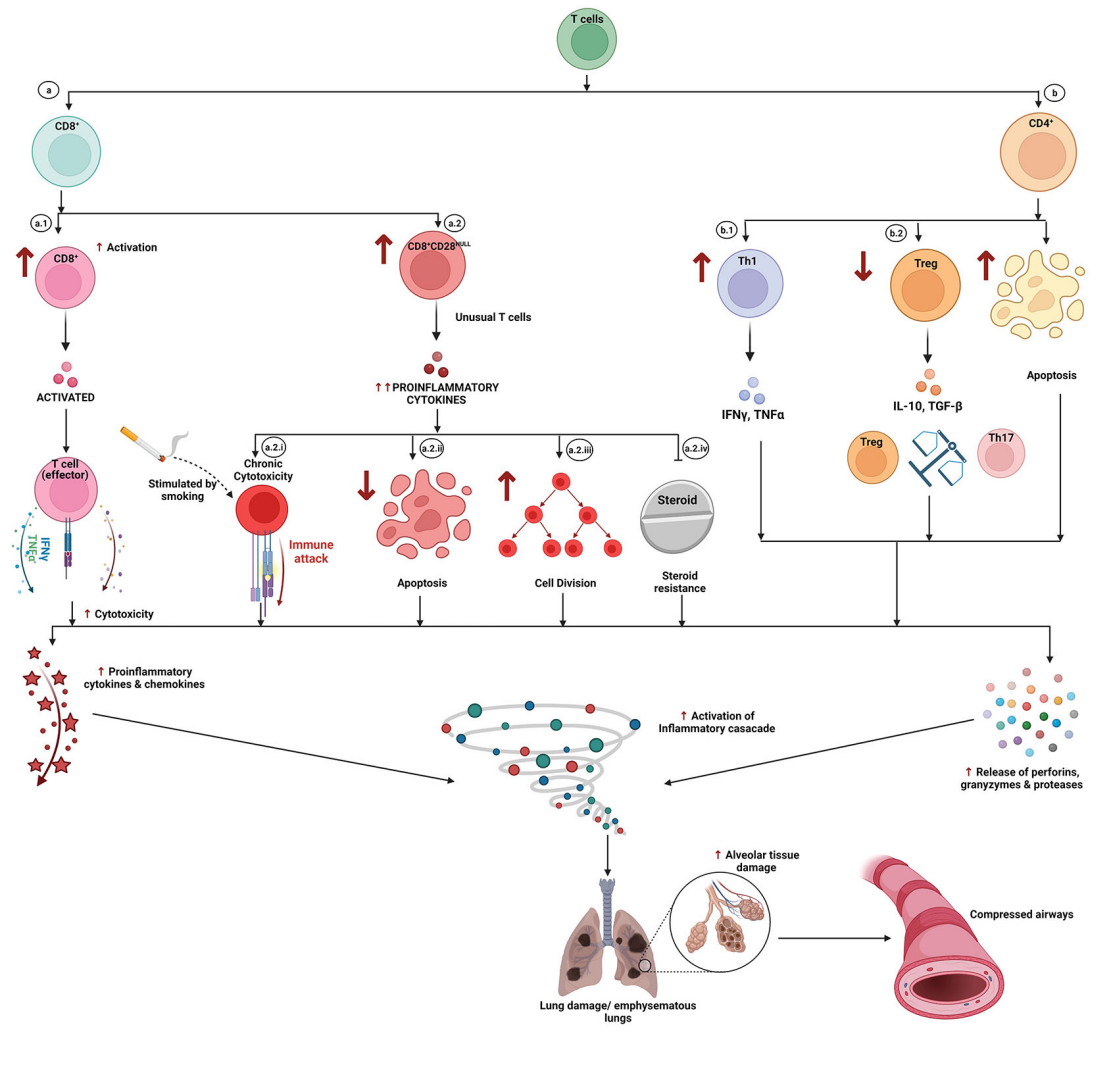
Role of lymphocytes in chronic obstructive pulmonary disease (COPD). T lymphocytes have been reported to play a vital role in the pathogenesis and progression of COPD. Exposure to allergens, respiratory tract infections, and cigarette smoking generate an inflammatory microenvironment that triggers a) increased activation of the CD8 ^+^ T cells (a1) and increased differentiation of the unusual CD8 ^+^CD28 ^NULL^ T cells (a2) and b) the increased differentiation of CD4 ^+^ cells to Th17 cells, decreased generation/differentiation of the immunoregulatory Treg phenotype (b1) and increased resistance to apoptosis (b2). a2) CD8 ^+^CD28 ^NULL^ T cells are characterized by exaggerated secretion of inflammatory mediators. a.2.i) These are permanently differentiated cytotoxic cells. a.2.ii) These cells have heightened expression of anti-apoptotic proteins, making the cells resistant to apoptosis (prolonged survival). a.2.iii) These cells manifest an increased rate of cell division. a.2.iv) These cells show resistance to the action of steroids. b1) Perturbation of the Th17 and Treg cell equilibrium along with b2) the resistance of these cells to apoptosis further exaggerates the proinflammatory microenvironment. a+b) Induces an inflammatory storm via exaggerated release of proinflammatory chemokines, cytokines, prostaglandins, reactive oxygen species, and cytotoxic enzymes like granzymes, perforins, and proteases. These promote alveolar tissue damage and intensify sterile lung inflammation as well as damage due to colonization by pathogenic microorganisms which culminates in widespread damage triggered compression/collapse of airways, characteristic of acute exacerbations or chronic long-term COPD. Note: ↑ indicates increased levels while ↓ refers to decreased levels/concentrations. This figure is an original figure produced by the authors for this review article and has been filed for copyright with the Copyright Office, Government of India.

➢ **Higher turnover and longer lifespan (** **Figure 6** **):** CD8 ^+^CD28 ^null^ clones divide faster and live longer than CD8 ^+^CD28 ^+^ T cells due to a shorter cell division cycle, higher resistance to apoptosis, and a different response to regulatory cytokines. ^140^
➢ **Steroid Resistant CD8** ^**+**^ **CD28** ^**null**^ **T Cells (** **Figure 5** **,** **6** **):** Acquired resistance to steroids (corticosteroid treatment) has emerged as a major challenge in the effective treatment of many chronic inflammatory conditions, especially COPD. Studies have reported elevated concentration (blood, BAL [bronchoalveolar lavage] & lungs) of CD8 ^+^CD28 ^null^ T cells in COPD patients (smokers as well as past smokers) regardless of whether patients are on inhalational corticosteroids or not. This indicates a blunted/sub-optimal effectiveness of steroids in blocking the chronically activated inflammatory cascades. ^141^

**Multiple Mechanisms/Causes of steroid resistance have been hypothesized and elucidated which include:**

• **Increased expression of P-glycoprotein-1 (Pgp1) (** **Figure 5** **):** P-glycoprotein, a transmembrane drug efflux protein pump (implicated in multi-drug resistant cancers), is upregulated in steroid-resistant CD8 ^+^CD28 ^null^ T and NK (natural killer) T cells in COPD patients, Enhanced P glycoprotein expression could lead to a relative steroid resistance. Further, studies have reported increased percentages of both CD8 ^+^Pgp1 ^+^CD28 ^null^ NKT-like and CD8 ^+^Pgp1 ^+^CD28 ^+^ NKT-like cells in COPD patients when compared to controls while treatment with very low-dose cyclosporine A (Pgp1 inhibitor) combined with standard-dose corticosteroid showed synergistic inhibition of pro-inflammatory cytokines. ^141^
• **Loss of glucocorticoid receptor (** **Figure 5** **):** Glucocorticoids must bind to the glucocorticoid receptors in the cytoplasm of a cell before being transported to the nucleus. Studies have reported a significantly diminished expression of glucocorticoid receptors in CD8 ^+^CD28 ^null^ T cells when compared to CD8 ^+^CD28 ^+^ T cells in COPD. ^142^^,^^143^
• **Decreased histone deacetylase (HDAC) expression (** **Figure 5** **):** HDACs are enzymes that regulate the transcription of inflammatory cytokines. Corticosteroids require HDAC2 to switch off activated inflammatory genes. Studies have shown diminished HDAC2 expression in CD8 ^+^CD28 ^null^ T cells of COPD patients. Sirtuin 1 (class III NAD-dependent histone deacetylase involved in silencing transcription of genes that produce inflammatory cytokines) expression is also reported to be diminished in the CD8 ^+^CD28 ^null^ T cells of COPD patients and has been postulated to be associated with steroid resistance, increased secretion of proinflammatory cytokines and disease severity. The study further reported that in presence of sirtuin 1 activator, sirtuin expression as well as steroid sensitivity of these cells can be restored. ^141^^,^^144^
• **Decreased heat shock protein 90 (** **Figure 5** **):** Hsp70 and Hsp90 are necessary for the acquisition of a high-affinity steroid binding conformation required for successful transport to the nucleus, but a recent study revealed that CD8 ^+^CD28 ^null^ T cells in COPD patients lack expression of both Hsp70 and glucocorticoid receptor. ^141^
➢ **Increased expression of alternate costimulatory surface markers (** **Figure 6** **):** Studies have reported that the loss of CD28 membrane receptors in CD8 ^+^CD28 ^null^ T cells may be compensated by an increased expression of alternate stimulatory receptors like CD137, OX40, CTLA4, etc which might play a role in the chronic activated proinflammatory state of these CD8 ^+^CD28 ^null^ T cells. ^140^
➢ **Cigarette smoking/activation of autoimmune component (** **Figure 6** **):** Cigarette smoking is postulated to initiate a self-maintaining pathogenic process that inhibits resolution. Studies have reported reduced CD28 expression in BALF CD8 ^+^ T cells among smokers. A study reported that COPD patients who quit smoking surprisingly showed increased circulating concentrations of CD8 ^+^CD28 null T cells. ^145^^–^^148^
  Studies are exploring the relevance of an activated autoimmune response in the pathophysiology and progression of COPD. It is hypothesized that a primary immune response to smoking gradually becomes self-perpetuating (stimulated by endogenous autoantigens resulting from inflammatory and oxidative lung injury and/or chronic respiratory tract infections). Evidence points towards the possibility of a loss of tolerance to self-antigens, or developments of immunity to foreign epitopes that cross-react with self-antigens in COPD with cigarette smoking/repetitive lung injury. These mechanisms are hypothesized to play a prominent role by generating new epitopes by oxidizing existing proteins in COPD. ^140^^,^^147^^,^^148^

**4.B.i.II) CD4+ T lymphocytes:**

**a) Activation of CD4** ^**+**^ **cells:** Studies have reported an increased concentration of CD4 ^+^ T lymphocytes in the airways and lung tissue of COPD patients as well as an increased proportion of activated CD4 ^+^ cells relative to non-COPD patients. ^132^^,^^149^ A study reported the presence of fully activated forms of both CD4 ^+^ and CD8 ^+^ T lymphocytes manifesting a predominant Th1/cytotoxic phenotype resulting in an exaggerated expression of proinflammatory and tissue injury cytokines. ^150^

**b) Apoptosis (** **Figure 6** **):** Exaggerated lymphocyte apoptosis is one of the most explored perturbations studied in COPD. Studies have reported enhanced overall T lymphocyte apoptosis as well as CD4 ^+^ T lymphocyte apoptosis in stable COPD patients while patients with acute exacerbations showed comparable apoptosis of both CD4 ^+^ and CD8 ^+^ subtypes. ^131^

**c) Blunted Treg response (** **Figure 6** **):** Treg cells, the immunosuppressive subset of CD4 ^+^ T cells, play an indispensable role in the maintenance of peripheral immune tolerance ^150^^,^^151^ Studies have reported lower Tregs in COPD patients when compared to healthy controls and blunted Treg cell response in COPD patients with a history of tobacco smoking. Further, healthy smokers (preserved lung function) showed a prominent upregulation of Tregs when compared to non-smokers. The upregulation in smokers with normal lung function may be interpreted as an attempt to regulate and minimize the inflammatory response elicited by tobacco smoking, whereas the failure of this mechanism in patients with COPD may contribute to the enhancement and/or dysregulation of such an inflammatory response, thus contributing to the pathogenesis of the disease. ^151^^–^^153^

**d) Th17/Treg imbalance (** **Figure 6** **):** The typical balance between Th17 and Treg T cell subsets is altered in various autoimmune diseases. ^154^ Th17 cells, a subset of CD4 ^+^ T cells, characterized by secretion of pro-inflammatory IL 17, are implicated to exert pivotal effects in many chronic inflammatory pathophysiologies. Both the cell lines develop from the common naive T CD4 ^+^ subset but functionally antagonize each other and maintain the critical balance of immune homeostasis. ^155^ Studies have reported an increased proportion of circulating Th17 cells in COPD patients. ^152^^,^^156^^–^^158^ A study reported a perturbed Th17/Treg balance in COPD with a shift towards pro-inflammatory response in patients showing acute exacerbations. ^152^ Another study found that a Th17/Treg imbalance existed in the bronchoalveolar lavage fluid of mice after chronic cigarette smoke exposure. ^156^

**4.B.ii) Role of B lymphocytes:**

Studies on inflammatory mechanisms involved in COPD focus majorly on exploring the role of T lymphocytes while the role of B lymphocytes remains to be elucidated. ^159^ A recent study has focussed on B lymphocytes and has reported markedly elevated lymphoid follicles (with B cells) in small airways of patients with advanced COPD when compared to mild COPD patients. ^160^ A study explored B cells aggregates from large airways and reported that B cell staining was significantly higher in COPD patients with higher counts in ex-smokers than in current smokers while another study reported the presence of B cell follicles in the lung parenchyma of COPD patients with predominance of CD27 ^+^ memory B cells. ^161^ The role of B cells in the pulmonary infiltrate of COPD patients remains unclear, it is hypothesized that these cells respond to chronic antigen stimulation by infectious agents and cigarette smoking. ^147^^,^^148^^,^^159^^,^^162^ B lymphocytes are the least explored immune cell type in COPD and further studies are required to identify their role in the pathophysiology of airway inflammatory diseases. ^159^

### 4. C) NLR in COPD

NLR, an emerging novel marker of systemic inflammation is being recently explored for its diagnostic and prognostic potential in COPD. ^163^ A study reported that NLR was increased in COPD patients when compared to controls and this increase was more pronounced in acute exacerbations of COPD, the authors concluded that NLR showed a significant association with smoking status, the severity of COPD, and in hospital mortality. ^164^ Another study reported similar findings and showed that NLR levels showed a positive correlation with CRP and a negative correlation with MPV (mean platelet volume). ^165^ A study on the Japanese population reported that NLR correlated with CRP at baseline and proposed a cut off of 2.7 to be a reliable indicator of severity and future exacerbations. ^166^ Another study reported that an NLR of 7.3 was an effective cut off to detect acute exacerbations of COPD. ^167^ A systematic review including 5140 patients reported that high NLR was associated with increased risk of exacerbation, short and long-term mortality and had a higher predictive ability in the Asian population. ^168^ Another systematic review including 7,601 COPD patients reported that NLR was significantly elevated in patients when compared to healthy controls, showed a significant correlation with pulmonary function, and proposed a cut off of 3.3 to diagnose acute exacerbations and a cut off of 7.3 to diagnose bacterial infections in exacerbated patients. ^169^

## Conclusion

Dissecting the myriad roles of NLR in chronic inflammatory diseases will provide a valuable framework for the development of treatments that can effectively bring about a balanced host inflammatory response and thereby prevent further disease progression. The current immunological context determines that the effects of altered NLR are diverse and may be either protective or harmful. NLR is increased by the activity pro-inflammatory cascades triggered by external factors, allergens, or endogenous inflammatory mediators and act as chronic activators of the immune response even in sterile microenvironments contributing to the susceptibility as well as the progression of chronic diseases such as type 2 diabetes, coronary artery disease, and chronic obstructive pulmonary disease. Therefore, pharmacological modulation of these pathways may present new therapeutic strategies for the management of chronic inflammatory diseases. In conclusion, NLR indicates disease severities, aids in the assessment of clinical outcomes, and offers potent novel therapeutic targets which could aid in the effective management of progressive chronic inflammatory lifestyle diseases.

## Data availability

No data are associated with this article.
