# Supplementary material for: The mechanistic role of neutrophil lymphocyte ratio perturbations in the leading non communicable lifestyle diseases
Source: F1000Res. 2022 Aug 19;11:960. [Version 1] doi: 10.12688/f1000research.123245.1 (PMC9780608; doi:10.12688/f1000research.123245.1)
Supplement: Peer review history

# Peer review: Reviewer response for version 1

**DOI:** 10.5256/f1000research.135331.r148012

**Published:** 2022-12-22

**Authors:** Prashant M. Vishwanath

**Affiliations:** 1 Center for Medical Genomics & Counselling, Department of Biochemistry, JSS Medical College, JSS Academy of Higher Education and Research, Mysore, Karnataka, India

The manuscript is well-written. However, there could be a few more points of discussion with regard to the action of myeloperoxidase enzyme and how it affects the neutrophils. MPO is released on neutrophil activation and binds to ECM and converts chloride anions plus hydrogen peroxide to hypochlorous acid. There is immunohistochemical evidence for the presence of MPO and HClO-modified proteins in plaques that have ruptured. The HClO at the sites of inflammation are known to induce apoptosis in endothelial cells. MPO further promotes survival of neutrophils by ligation of CD18 thereby prolonging the inflammation. Further, the roles of MMP-2 and MMP-9 can be elaborated along with the action of neutrophil elastase.

**Objective:** To understand the pathological mechanisms that trigger NLR perturbations in non-communicable diseases i.e. type-2 Diabetes mellitus, Coronary artery disease and Chronic obstructive pulmonary disease.

 The neutrophil lymphocyte ratio (NLR) indicates the balance between systemic inflammation and immunity. NLR can be considered as an indicator of systemic inflammation. As neutrophils and lymphocytes are the cells included in the White Blood Corpuscles population, which are responsible for protecting our body from infections, their count or their ratio (NLR ratio) can be considered as one of the biomarkers for chronic inflammation.

**1.Type 2 Diabetes**

**a) Neutrophils**

- In hyperglycaemia, there is an increase in intracellular cAMP levels, which in turn increases the neutrophil production and activation.
- Hyperglycaemia also triggers the formation of Advanced Glycated End products (AGEs) and the release of acute phase proteins which in turn causes increased neutrophil activation.
- The increase in TNF α increases the neutrophil production and activation, and there occurs a resistance to apoptosis and dysfunctional chemotactic and phagocytic abilities, which finally leads to micro and macro vascular complications of Diabetes mellitus.

**b) Lymphocytes**

- There occurs an insufficient lymphocyte proliferation due to lower expression of IL-2.
- Low lymphocyte concentration and high neutrophil concentration can be observed.
- Hyperglycaemia induces stress and oxidative damage in T-lymphocytes.

**2. Coronary Artery Disease (CAD)**

**a) Neutrophils**

- Hyperlipidaemia and oxidative damage create a chronic low-grade inflammatory microenvironment that activates the release of granulocyte-colony stimulating factor (GCSF) and proinflammatory mediators like IL-6, TNF α etc.
- GCSF triggers the proliferation of neutrophils and IL-6, TNF α retards neutrophil apoptosis.
- There occurs a decrease in CXCL12, which triggers enhanced neutrophil mobilization. Finally, the entire sequence leads to the plaque rupture and thrombosis that causes myocardial infarction.
- Neutrophil Extracellular Traps formation (NETosis) affects the inflammation process (might be explained ****clearly).

**b) Lymphocytes**

- Hyperlipidaemia creates a chronic low-grade inflammatory microenvironment which in turn triggers the increased differentiation of CD4+ cells to Th and Th17 phenotypes.
- Chronic inflammation triggers generation of CD4+ cells, CD28 null T-cells, then those cells increase the expression of CXCR1 which results in the expression of BCL2 anti-apoptotic protein.
- There occurs an increased expression of BAFF, which aids B2 cell maturation and causes resistance to apoptosis.

**3. Chronic Obstructive Pulmonary disease**

**a) Neutrophils**

- When we are exposed to allergens or smoke, it generates an inflammatory microenvironment, the increased immune cell migration occurs which in turn increases the secretion of TNF α, which stimulates neutrophil production, proliferation, and differentiation.
- Here, increased neutrophil activation and increased resistance to apoptosis also occur which further results in compressed airways and defective mucociliary clearance.

**b) Lymphocytes**

- Exposure to allergens, cigarette smoking etc. generate an inflammatory microenvironment that triggers activation of CD8+ T cells, which in turn leads to the increased differentiation of CD4+ and CD28+ null cells, then there occurs an increase in resistance to apoptosis due to the release of anti-apoptotic proteins.

- Is the topic of the review discussed comprehensively in the context of the current literature? **The topic in review, "Mechanistic Role of NLR perturbations in the leading non communicable lifestyle diseases", is discussed comprehensively in the current context and recent literature has been cited.**
- Are all factual statements correct and adequately supported by citations? **The factual statements are correct and adequately supported by citations.**
- Is the review written in accessible language? **The review has been written in accessible language so that every reader should be able to understand.**
- Are the conclusions drawn appropriate in the context of the current research literature? **The conclusions drawn are appropriate and the suggestions have been mentioned in the peer review report.**

Is the review written in accessible language?

Yes

Are all factual statements correct and adequately supported by citations?

Yes

Are the conclusions drawn appropriate in the context of the current research literature?

Yes

Is the topic of the review discussed comprehensively in the context of the current literature?

Yes

Reviewer Expertise:

Non communicable diseases, metabolic disorders, molecular biology and biochemistry, immunology

I confirm that I have read this submission and believe that I have an appropriate level of expertise to confirm that it is of an acceptable scientific standard.

# Peer review: Reviewer response for version 1

**DOI:** 10.5256/f1000research.135331.r148009

**Published:** 2022-10-27

**Authors:** Vivek R. Joshi

**Affiliations:** 1 Department of Biochemistry and Molecular Biology, Drexel University College of Medicine, Philadelphia, PA, USA

Thank you for providing me with this opportunity to review this article for your esteemed journal.

 Here are my thoughts on the article.

1. The topic of the review is important and interesting and is discussed comprehensively by the authors.
2. Authors have provided relevant references to the literature reviewed.
3. The article is written well, appropriate use of language, grammar, and syntax.
4. The conclusion drawn is relevant and interesting, it can be elaborated further to include the other pertinent findings in the review.

Changes recommended:

1. Kindly mention the type of review as it is not a structured or systematic review.  
2. Conclusion can be elaborated more to justify the review. 

I am looking for a slightly detailed conclusion. The conclusion in present form is acceptable but it has an opportunity to summarize and narrate all the discussion points into conclusion. This part seems to be deficient but, by including a few more points, it will make it better.

 Regarding the conclusion: There is a grammatical error in line 4. The authors can elaborate the conclusion by describing the various therapeutic strategies utilized to manage the chronic inflammatory diseases, what could be the potent therapeutic targets used based on the review.

Is the review written in accessible language?

Yes

Are all factual statements correct and adequately supported by citations?

Yes

Are the conclusions drawn appropriate in the context of the current research literature?

Yes

Is the topic of the review discussed comprehensively in the context of the current literature?

Yes

Reviewer Expertise:

Clinical biochemistry, chemical pathology, endocrinology

I confirm that I have read this submission and believe that I have an appropriate level of expertise to confirm that it is of an acceptable scientific standard.
